# Artificial intelligence based prediction of first recurrence in neovascular age related macular degeneration with validation by 19 experts

**DOI:** 10.1038/s41598-025-34480-8

**Published:** 2026-01-16

**Authors:** Boa Jang, Chan Ho Lee, Seung Jin Kim, Chang Ki Yoon, Un Chul Park, Jinwook Choi, Eun Kyoung Lee, Young-Gon Kim

**Affiliations:** 1 Interdisciplinary Program in Bioengineering, College of Engineering, Seoul National University, Seoul, Republic of Korea; 2 Department of Transdisciplinary Medicine, Seoul National University Hospital, #101, Daehak-Ro, Jongno-Gu, Seoul, 03080 Republic of Korea; 3 Department of Ophthalmology, Seoul National University College of Medicine, Seoul National University Hospital, #101, Daehak-Ro, Jongno-Gu, Seoul, 03080 Republic of Korea; 4 Department of Biomedical Engineering, College of Medicine, Seoul National University, Seoul, Republic of Korea; 5 Institute of Medical and Biological Engineering, Medical Research Center, Seoul National University, Seoul, Republic of Korea; 6 Department of Medicine, Seoul National University College of Medicine, Seoul, Republic of Korea; 7 Healthcare AI Research Institute, Seoul National University Hospital, Seoul, Republic of Korea

**Keywords:** Neovascular age-related macular degeneration, Artificial intelligence, Ophthalmologist, Optical coherence tomography, Recurrence prediction, Medical imaging, Tomography, Randomized controlled trials

## Abstract

This study aimed to investigate the value and difference in predictive performance between ophthalmologists and a previously developed and validated artificial intelligence (AI) model, and to evaluate how AI assistance influences expert decision-making in reliably assessing recurrence prediction of neovascular age-related macular degeneration (nAMD) after anti-vascular endothelial growth factor (VEGF) treatment. 19 experts (nine retinal specialist ophthalmologists and ten non-retinal specialist ophthalmologists) predicted the first recurrence of nAMD within three months based on optical coherence tomography (OCT) images and clinical information. Predictions were made in five sessions with increasing information availability. The AI model used in this study had been developed and validated in our earlier work, and it predicted recurrence using baseline and after the loading phase OCT images. We compared the area under the receiver operating characteristic curve (AUROC), Fleiss’ kappa, and Delong’s test between expert groups and the AI algorithm. The study included 149 eyes of 130 patients. The AI model achieved an AUROC of 0.744 (95% confidence interval, 0.665–0.822). Expert performance improved across sessions, with AUROCs ranging from 0.562 ± 0.034 to 0.679 ± 0.049. No significant differences were observed between expert groups based on experience or subspecialty. AI-supported decisions showed slightly improved performance in predicting nAMD recurrence compared to human experts, regardless of clinical experience. These results suggest the potential of AI-assistance in predicting recurrence and optimizing treatment strategies for nAMD, which could significantly improve patient counseling and management. This study also highlights the novel contribution of evaluating the impact of AI assistance on ophthalmologists’ decision-making in nAMD recurrence prediction.

**Supplementary Information:**

The online version contains supplementary material available at 10.1038/s41598-025-34480-8.

## Introduction

Neovascular age-related macular degeneration (nAMD) is a major cause of vision loss worldwide^1,2^ wherein increased levels of vascular endothelial growth factor (VEGF) lead to neovascularization of the choroidal and/or retinal vasculature^3^. Neovascular leakage causes the accumulation of pathological retinal fluids such as the intraretinal fluid (IRF), subretinal fluid (SRF), or pigment epithelial detachment (PED) in the macular area and damages the neurosensory retina^4^. Inhibition of VEGF by the intravitreal injection of anti-VEGF antibodies allows for fluid reduction and disease stabilization. Accordingly, anti-VEGF therapy is now the established treatment of choice for nAMD, preserving vision in many patients, and clinicians focus on fluid changes in optical coherence tomography (OCT) images to determine the treatment strategy^5^.

However, the short duration of action of anti-VEGF agents requires persistent treatment. Disease activity and the interval between recurrences are highly heterogeneous among patients, making treatment decisions challenging for clinicians. Understanding when the first recurrence occurs after three consecutive anti-VEGF loading treatments would be beneficial to clinicians in these decision-making challenges. Predicting the first recurrence can help decide how often to follow up patients after the loading phase and administer anti-VEGF therapy.

An artificial intelligence (AI)-based computer-aided diagnosis (CADx) system has been developed to predict nAMD progression using OCT. The latest CADx systems employ deep learning (DL) techniques, which are state-of-the-art technologies that deliver ideal outcomes^6–10^. Treder et al.^7^ utilized the InceptionV3^11^ model to differentiate between nAMD and normal OCT images. Hwang et al.^9^ utilized an AI-based cloud platform that accurately diagnosed nAMD and suggested treatment strategies, demonstrating its potential for use in telemedicine. However, previous studies have primarily focused on diagnosing nAMD in comparison with normal cases, rather than predicting the prognosis of nAMD.

Building on our preliminary study, which developed a DL model utilizing OCT images to predict whether the first recurrence would occur within three months after the loading phase, we demonstrated the feasibility of the algorithm^12^. Although the task of predicting recurrence using only OCT images was very challenging, even for the advanced DL model, the performance was higher when using after-loading-phase OCT than baseline OCT images.

**Fig. 1:**
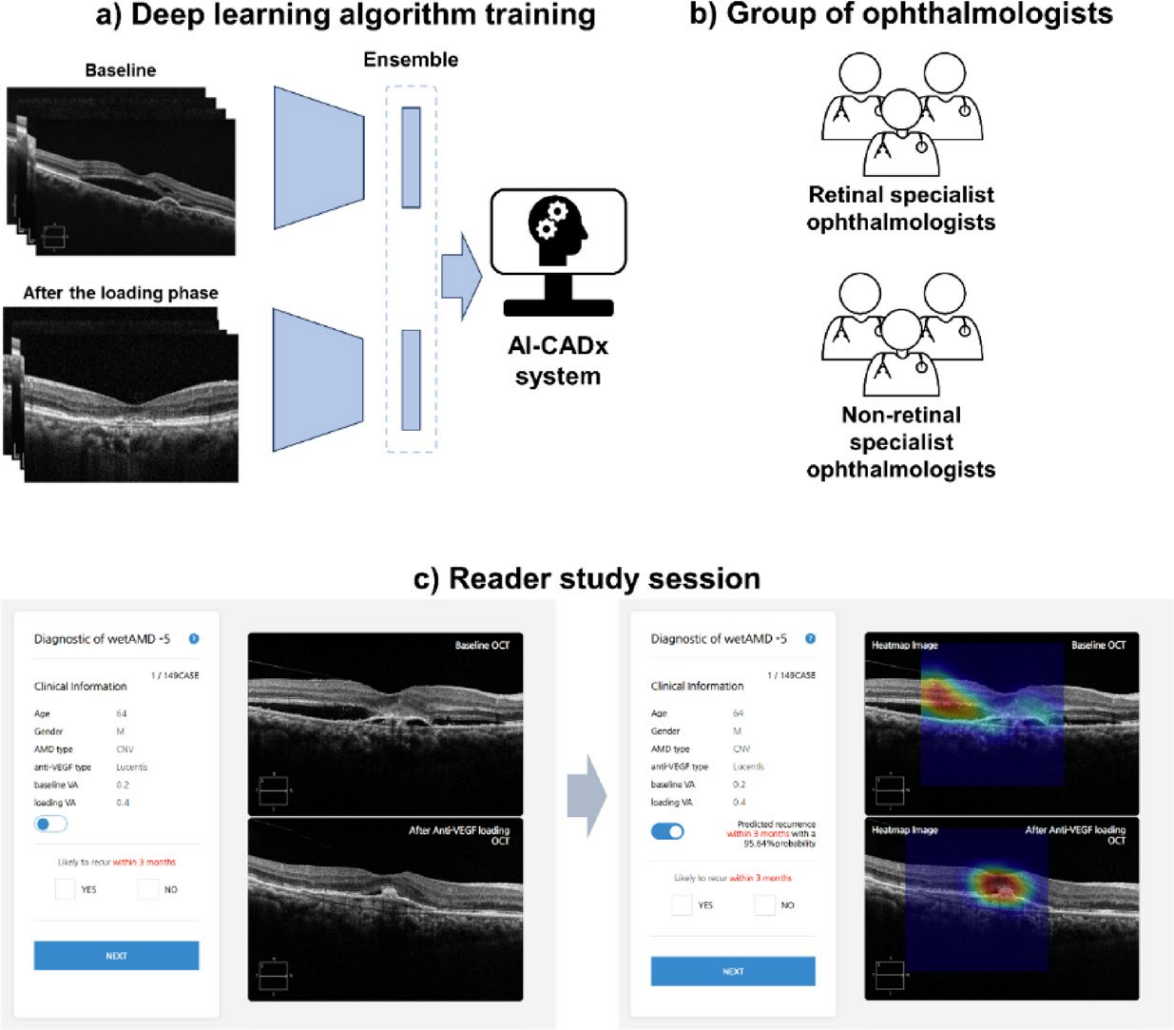
Schematic diagram of the reader study workflow. (**a**) training workflow of artificial intelligence-based computer-aided diagnosis system; (**b**) two groups of ophthalmologists involved in this study; (**c**) overview of reading sessions.

In this study, we aimed to extend our previous work by directly comparing the predictive performance of a previously validated DL model with that of ophthalmologists in forecasting the first recurrence of nAMD using OCT images. The overall workflow of the reader study is illustrated in Fig. 1, and participating ophthalmologists with varying subspecialties and experience levels completed five sequential reading sessions with increasing information availability, as shown in Supplementary Fig. 1. Beyond simple performance comparison, this study also sought to evaluate how AI-generated recurrence scores and heatmaps influence expert decision-making and inter-reader consistency across these sessions, thereby clarifying the potential role of AI assistance in clinical prediction of nAMD recurrence.

## Results

### Characteristics of the participants

A total of 149 eyes of 130 patients in the test set were evaluated in the current study (Table 1). Among the 149 eyes, 77 (51.7%) showed recurrence within 3 months and 72 (48.3%) experienced recurrence after 3 months, indicating a relatively balanced class distribution. The mean age of the patients was 72.1 ± 8.1 years. No significant differences were noted in the laterality, baseline best-corrected visual acuity (BCVA) in the logarithm of the minimum angle of resolution (logMAR), axial length, nAMD subtype, or anti-VEGF agents used for the loading phase between the two groups when the first recurrence was within 3 months after the loading phase. The first recurrence within 3 months group had a higher proportion of male participants and exhibited worse BCVA after the loading phase.

**Table 1:** Demographics and baseline clinical characteristics of the study participants.

Variables	Recurrence within 3 months	Recurrence after 3 months	Total	*p*-value
Number of samples	77	72	149	
Age at first nAMD diagnosed (yrs)	72.17 ± 7.97	71.93 ± 8.19	72.1 ± 8.10	0.857*
Sex				0.029^†^
Male	51 (34.2)	34 (22.8)	85 (57.0)	
Female	26 (17.4)	38 (25.5)	64 (43.0)	
Laterality				0.290^†^
Right eye	43 (28.9)	33 (22.1)	76 (51.0)	
Left eye	34 (22.8)	39 (26.2)	73 (49.0)	
BCVA (logMAR)				
At baseline	0.78 ± 0.53	0.74 ± 0.49	0.76 ± 0.51	0.608*
After the loading phase	0.68 ± 0.52	0.48 ± 0.40	0.58 ± 0.47	0.010*
Axial length (mm)	23.86 ± 1.21	23.39 ± 0.77	23.62 ± 1.03	0.071*
nAMD subtype				0.192^†^
Type 1 or 2 CNV	64 (42.95)	51 (34.23)	115 (77.18)	
PCV	7 (4.70)	10 (6.71)	17 (11.41)	
RAP (Type 3 CNV)	6 (4.03)	11 (7.38)	17 (11.41)	
Anti-VEGF used for loading phase				0.236^†^
Ranibizumab	43 (28.86)	31 (20.81)	74 (49.67)	
Aflibercept	27 (18.12)	35 (23.49)	62 (41.61)	
Bevacizumab	7 (4.70)	6 (4.03)	13 (8.73)	

BCVA, best-corrected visual acuity; CNV, choroidal neovascularization; PCV, polypoidal choroidal vasculopathy; logMAR, logarithm of the minimum angle of resolution; nAMD, neovascular age-related macular degeneration; RAP, retinal angiomatous proliferation.

Continuous variables are reported as mean value ± standard deviation. All other data are presented as numbers (percentages).

*Student independent t-test.

^†^Chi-square test or Fisher exact test.

Twenty ophthalmologists were recruited for the study, of which ten were retinal specialist ophthalmologists (RSOs) and ten non-retinal specialist ophthalmologists (N-RSOs) (Supplementary Table 1). One retinal specialist ophthalmologist was excluded due to incomplete readings, resulting in 19 readers in the final analysis. Based on these 19 participants, the average age was 35.0 ± 5.61 years, and the average years of experience was 8.47 ± 6.20 years.

### Comparison between AI-unassisted and AI-assisted reading performance

The performance evaluation of the AI-based CADx system using our in-house validation dataset, as previously reported in our earlier work^12^, revealed an area under the receiver operating characteristic curve (AUROC) score^13^ of 0.600 (95% CIs 0.568–0.743) for OCT images at baseline and an AUROC of 0.725 (95% CIs 0.658–0.817) for OCT images after the loading phase^12^. The ensemble of the two models through hard voting resulted in an improved AUROC of 0.744 (95% CI 0.655–0.822) for image-wise classification. The ensemble of baseline and after the loading phase OCT models achieved an accuracy of 0.698, sensitivity of 0.571, specificity of 0.833, positive predictive value (PPV) of 0.786, negative predictive value (NPV) of 0.645, and an F1 score of 0.622. These metrics provide a more comprehensive assessment of the model’s predictive capability, and the full details are provided in Supplementary Table 2.

Supplementary Fig. 2 depicts the performance metrics of all experts over the five reading sessions. A significant increase was noted in the AUROC score, reaching 0.679 in session 5, and 0.562 (*p* < 0.001), 0.663 (*p* < 0.05), and 0.649 (*p* < 0.001) in sessions 1, 3, and 4, respectively. The AI-based CADx system led to notable improvements in the AUROC across sessions 1, 3, and 4 (*p* < 0.05). Notably, session 2, which relied exclusively on OCT images after the loading phase for prognostic prediction, demonstrated outcomes comparable to those of session 5 (*p* = 0.307), as shown in first column of Table 2.

**Table 2:** Performance of nine RSOs and ten N-RSOs in five reading sessions.

Session	Total AUROC [95% CIs]	*p*-value	RSOs AUROC [95% CIs]	*p*-value	N-RSOs AUROC [95% CIs]	*p*-value	RSOs vs. N-RSOs
1	0.562*** ± 0.034 [0.545, 0.578]	< 0.001	0.554** ± 0.039 [0.522, 0.586]	< 0.01	0.568*** ± 0.027 [0.548, 0.589]	< 0.001	n.s
2	0.665 ± 0.044 [0.644, 0.687]	0.307	0.657 ± 0.040 [0.625, 0.690]	0.541	0.673 ± 0.046 [0.638, 0.708]	0.437	n.s
3	0.663* ± 0.051 [0.638, 0.687]	0.033	0.658 ± 0.065 [0.606, 0.711]	0.329	0.667 ± 0.033 [0.642, 0.692]	0.053	n.s
4	0.649*** ± 0.054 [0.623, 0.675]	< 0.001	0.642* ± 0.073 [0.583, 0.702]	0.020	0.655** ± 0.027 [0.635, 0.676]	0.005	*n.s*
5	**0.679** ± 0.049 [0.655, 0.702]	Ref	**0.670** ± 0.064 [0.618, 0.722]	Ref	**0.686** ± 0.026 [0.666, 0.706]	Ref	n.s

AUROC, area under the receiver operating curve; CIs, confidence intervals; RSOs, Retinal Specialist Ophthalmologists; N-RSOs, Non-Retinal Specialist Ophthalmologists.

*p*-values were calculated using Delong’s test. *p < 0.05, **p < 0.01, ***p < 0.001, n.s.: non-significant.

No *p*-value is provided for session 5 because it served as the AI-assisted reference session. Values in bold indicate the reference session (Session 5) used for comparison with other sessions.

### Comparative performance across different reader levels and inter-rater agreement between experts

Comparative performances across different reader levels and inter-rater agreement among different experts across the five reading sessions are highlighted in right side of Tables 2 and Supplementary Table 3, respectively. Table 2 also outlines the differences in performance between the RSOs and N-RSOs groups across the five reading sessions. Across sessions 1, 4, and 5, the AI-based CADx system significantly improved the AUROC scores for both groups (*p* < 0.05). For example, the overall AUROC increased from 0.562 (95% CI 0.545–0.578) in session 1 to 0.649 (95% CI: 0.623–0.675) in session 4 and 0.679 (95% CI 0.655–0.702) in session 5. Importantly, no statistically significant differences were observed between RSOs and N-RSOs in any session (*p* > 0.05), suggesting that the AI-based CADx system equally benefits both experienced retinal specialists and non-specialists in improving their ability to predict first recurrence.

Supplementary Table 3 details the reliability of agreement among different experts across the five reading sessions, quantified using Fleiss’ kappa to assess inter-rater agreements. The scoring convention for Fleiss’ kappa ranged from less than 0, indicating poor agreement, to slight, fair, moderate, substantial, and almost perfect agreement, with a score close to 1. It also highlights the improvement in the reliability of agreement among different experts with the AI-based CADx system across all sessions (*p* < 0.05), except for session 2 (*p* = 0.161). In session 5, the Fleiss’ kappa score indicated a moderate level of agreement, underscoring the impact of AI on enhancing prediction consistency among various experts. These results demonstrate how the AI-based CADx system aids in achieving more consistent prediction evaluations among different experts, particularly in sessions in which the kappa values reached statistical significance.

### Prediction agreement between experts

**Fig. 2:**
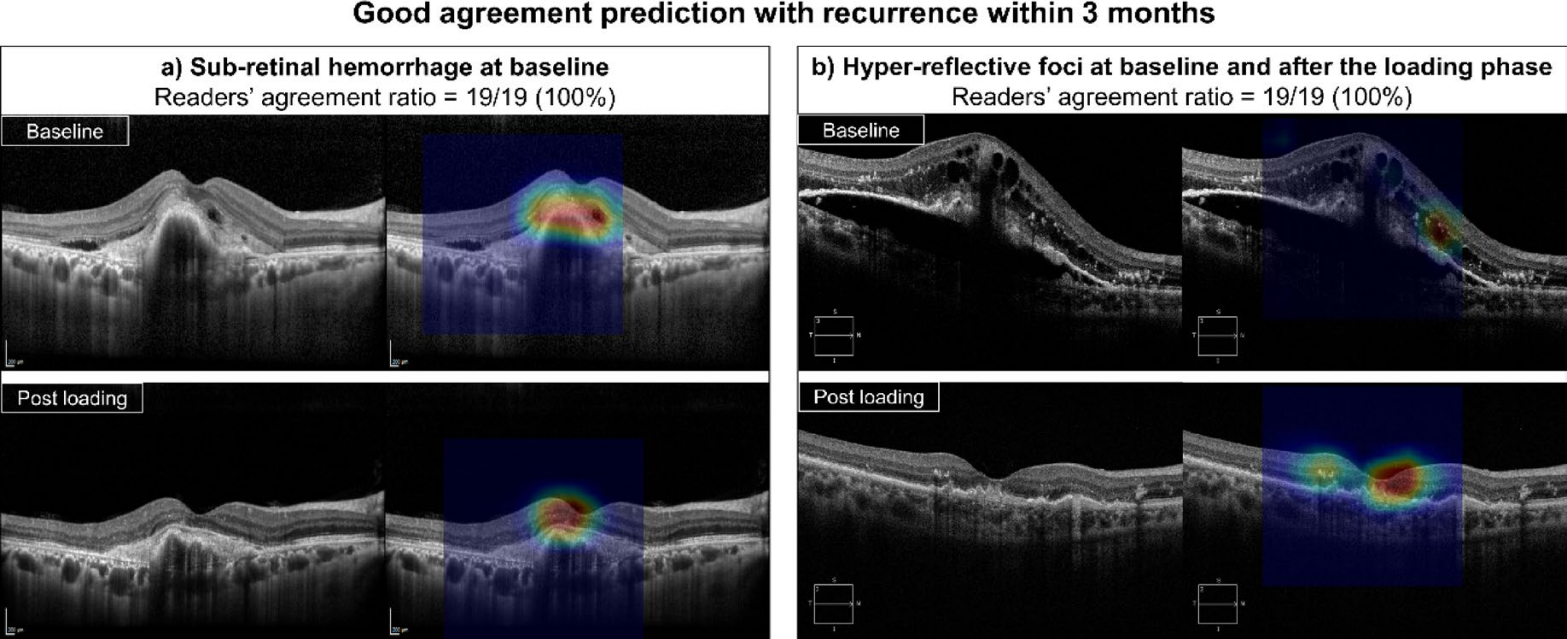
Representative cases of optical coherence tomography (OCT) images and gradient-weighted class activation mapping (Grad-CAM) visualization where an early recurrence was predicted with good inter-reader agreement. Subretinal hemorrhage at baseline (**a**) and hyperreflective foci at baseline and after the loading phase (**b**) were determined OCT features resulted in good agreement prediction with recurrence within 3 months between experts.

A subgroup analysis was performed to determine which OCT features resulted in better agreement between the experts. A prediction was considered “good” when more than 70% of experts made the same prediction regarding the time of first recurrence. The degree of agreement is a crucial metric for assessing the reliability of the predictions and guiding the implementation of AI-assisted decision-making processes. Supplementary Table 4 shows the results of the analysis of cases with good agreement between experts categorized by a time to the first recurrence of 3 months. Early recurrence was predicted by the experts with good agreement if there was baseline subretinal hemorrhage or intraretinal hyper-reflective foci at baseline or after the loading phase (*p* < 0.001). Figure 2 shows the representative cases in which early recurrence was predicted with good inter-expert agreement. When subretinal hemorrhage was present at baseline and intraretinal hyperreflective foci were observed at baseline and after the loading phase, the experts predicted early recurrence within 3 months with good agreement.

## Discussion

In this study, we conducted a comparative analysis of AI-assisted and non-AI-assisted recurrence predictions for nAMD, revealing significant insights into the role of AI in improving the ability to predict future recurrence. The AI-based CADx system consistently showed a notable improvement in performance across various experiments, underscoring the potential of the AI algorithm as a valuable tool for enhancing clinical decision making, irrespective of the specialist’s experience level. Specifically, the readings following the OCT images after the loading phase (session 2) aligned closely with those of the AI-based CADx system. This indicates that after the loading phase, OCT is an important factor in predicting the first recurrence of nAMD, further indicating that post-treatment morphological changes and clinical information are critical for predicting nAMD recurrence.

Statistically significant improvements in AUROC were observed in session 1 (*p* < 0.001), session 3 (*p* < 0.05), and session 4 (*p* < 0.001), whereas the sensitivity and specificity did not show significant differences in most sessions. Overall, AUROC was more stable and reliable than sensitivity or specificity, which showed substantial variability and rarely reached statistical significance, except for specificity in a few sessions. This indicates that although the AI-based CADx system may improve the overall accuracy of predictions, the consistency of detecting true positives (sensitivity) and true negatives (specificity) could vary significantly across different sessions.

Although the AUROC of 0.744 reflects moderate discriminative performance, this level of accuracy may still offer meaningful clinical value. In particular, a model capable of identifying patients with a higher likelihood of early recurrence can support triage and risk-stratification workflows after the loading phase. By flagging higher-risk individuals who may require closer monitoring or shorter follow-up intervals, the model could help prevent delayed detection of recurrent exudation, whereas lower-risk patients may be followed more flexibly. Therefore, even a moderately performing model may function as a practical adjunct to clinical decision-making rather than a standalone diagnostic tool.

However, when comparing the diagnostic performance between RSOs and N-RSOs, the comparative performance in the two groups revealed persistent difficulties in interpreting OCT images for predicting nAMD recurrence, despite the AI-based CADx system. This absence of performance difference highlights the inherent difficulty of predicting early nAMD recurrence from OCT images, even among experienced specialists. This challenge is also reflected in the broader literature. Previous studies primarily used machine learning to predict the demand or frequency of anti-VEGF treatment for nAMD^14–17^, rather than to directly forecast recurrence timing. However, studies focused specifically on predicting the timing of nAMD recurrence remain limited^12,18^. A recent study by Jung et al.^18^ proposed a DL model to predict whether patients with nAMD will experience their first recurrence within three months after receiving three loading injections and following one month of dry-up. They reported that the DL model achieved 53.0% accuracy in predicting nAMD recurrence using a single pre-injection image and 60.2% accuracy after viewing consecutive OCT images, outperforming ophthalmologists with 52.17% and 53.3% accuracy, respectively. They further found that both human specialists and the DL model showed limited ability to accurately predict outcomes based only on a single pretreatment OCT image, leading to almost random results. In our previous work, the DL model achieved an AUC of 0.725 using only a single after the loading phase OCT image^12^, whereas in the current study AI-assisted ophthalmologists achieved an AUC of 0.678. Although our model performed better than that of Jung et al.^18^, the collective evidence consistently demonstrates that predicting early nAMD recurrence using OCT images alone remains a fundamentally challenging task for both AI models and human experts.

Additionally, this study confirmed that the presence of a subretinal hemorrhage or hyperreflective foci at baseline plays an important role in determining whether recurrence should be predicted by ophthalmologists. Moreover, the presence of subretinal hemorrhages in nAMD releases iron and hemosiderin into the environment, causing oxidative stress and ongoing damage to the macula^19,20^, and this ongoing damage makes it more likely to recur. The presence of hyperreflective foci early in the disease has been reported to be associated with increased progression of nAMD^21^. In nAMD, hyperreflective foci have been reported to be present from the earliest stages of the disease, before anti-VEGF injections, and the greater the amount, the higher the markers of inflammation^22^. In our previous study, heatmap analysis of the recurrence classification mainly highlighted areas of pathological fluid, subsided choroidal neovascularization lesions, and hyperreflective foci on OCT scans^12^. In this study, the presence of subretinal hemorrhage and hyperreflective foci were important for ophthalmologists in predicting early recurrence, confirming the role of these OCT biomarkers in predicting recurrence timing.

This study found that AI had a significant impact on improving consistency and reducing discrepancies in diagnostic approaches by analyzing the reliability of agreement among different experts over five sessions. This suggests that AI may contribute to a more standardized interpretation of complex OCT images, which is an important aspect for reliable diagnostic results. Reviewing the predictive agreements among experts also provides valuable insights into the OCT parameters related to agreement among diagnosticians, which along with the AI-based CADx system, could contribute to the development of standardized diagnostic criteria while addressing the diversity of individual interpretations.

Despite these positive findings, this study had several limitations. First, the performance of the AI algorithm did not reach a superior level, owing to an AUROC score of 0.744, which did not reach the desired threshold. Second, the study was conducted at a single tertiary referral center, and no external validation with an independent dataset was performed, which may limit the generalizability of our findings to broader clinical settings. Additionally, the relatively small dataset and the heterogeneous anti-VEGF treatment regimens, as noted in our previous work, may introduce variability in ground-truth labeling and affect the robustness of both the AI performance and the human–AI comparisons. Third, the one-day interval between reading sessions provides a potential cause of bias, emphasizing the need for careful consideration of the study design in future investigations. Finally, because experts had access to both OCT images and clinical information whereas the AI model relied solely on OCT images, the comparison was not fully symmetric. Future models incorporating relevant clinical variables may allow a more balanced and comprehensive evaluation.

To address these limitations, future studies should focus on collecting additional data and model tuning to improve the performance of AI-based CADx systems. In particular, external validation using multi-center datasets will be essential to assess the model’s generalizability across diverse clinical environments. Developing a model for predicting the actual recurrence time is a crucial step forward, providing clinicians with more precise information for treatment planning. In addition, developing AI-based applications tailored for ophthalmology clinics has the potential to improve the diagnostic process, efficiency, accuracy, and consistency in clinical settings. These methods of future work aim to take advantage of AI while addressing its current limitations and ultimately increasing its productivity in the field of ophthalmology.

## Methods

### Deep learning algorithms

In this study, an AI-based CADx system using OCT was employed^12^. The model had been previously trained and validated using 1,295 OCT images from 1172 patients, with a balanced distribution between recurrence and non-recurrence cases. The dataset was randomly split into 70% training, 20% validation, and 10% test sets at the patient level, and five-fold cross-validation was performed to address the limited dataset size and reduce overfitting. The AI algorithm involves a dual-step process: first, it identifies the fluid regions via a fluid segmentation model, followed by a binary classification to predict the recurrence of nAMD within three months after the loading phase. These two distinct AI algorithms are subsequently integrated into a comprehensive AI-based CADx system. The system generates a recurrence score ranging from 0 to 100% that reflects the likelihood of recurrence within a specified period and also provides a heat map that highlights the primary regions prone to recurrence.

For reproducibility, we additionally summarize the key components of the previously developed AI model used in this study. Fluid regions were first localized using a U-Net–based segmentation module trained on expert-annotated OCT images, and the resulting fluid masks were used to extract fluid-centered patches (400 × 400 pixels) as classifier inputs. The recurrence classification network used a ResNet50 backbone pre-trained on ImageNet and was trained for 100 epochs using softmax cross-entropy loss, the Adam optimizer (learning rate 0.0001), and a batch size of 8. Final recurrence predictions were generated by combining outputs from classifiers trained on baseline and after the loading phase OCT images through a hard-voting ensemble, which served as the model presented to readers during the AI-assisted session.

To ensure the integrity of the AI model development, all dataset splits for training, validation, and testing were conducted at the patient level, such that both eyes from the same individual were assigned to the same subset. This prevented any leakage of patient-specific information across data partitions.

### Patients and datasets

This retrospective study included patients with treatment-naïve nAMD who visited Seoul National University Hospital (SNUH) between February 2008 and July 2021. All patients were treated with three consecutive loading intravitreal injections of ranibizumab (Lucentis; Novartis, Basel, Switzerland), aflibercept (Eylea; Bayer Pharma, Germany), or bevacizumab (Avastin; F. Hoffmann-La Roche Ltd., Basel, Switzerland). Baseline OCT scans were obtained when the patient was first diagnosed with nAMD, whereas OCT scans after the loading phase were taken one month after three consecutive loading injections. In this study, recurrence was defined as the initial appearance of a new retinal hemorrhage or intra/subretinal fluid accumulation after the initial resolution of exudative changes after three loading injections. Although the persistence of PED was not considered a recurrence, the increase in PED size was considered as recurrence. This study was approved by the Institutional Review Board of Seoul National University Hospital (IRB approval number: 2107-223-1239) and adhered to the tenets of the Declaration of Helsinki. The Institutional Review Board of Seoul National University Hospital waived the need for written informed consent from the participants because of the retrospective design of the study.

### Experiment setup

Twenty ophthalmologists were recruited to assess their performance. A comprehensive workflow of the experiment is shown in Fig. 1. The experiment consisted of five reading sessions, and each session was followed by a one-day washout period. The reading times were automatically recorded using a web-based in-house tool. The information provided in each session was as follows: patient’s baseline OCT only (session 1), patient’s after the loading phase OCT only (session 2), both the patient’s baseline and after the loading phase OCT (session 3), clinical information including age, sex, nAMD types, and anti-VEGF agents used, with both the patient’s baseline and after the loading phase OCT (session 4), DL algorithm results, clinical information, and both the patient’s baseline and after the loading phase OCT (session 5). In session 5, the AI algorithm outputted a recurrence score expressed as a percentage from 0 to 100, indicating the likelihood of the first recurrence of nAMD within 3 months after the loading phase. In addition, gradient-weighted class activation mapping (Grad-CAM)^23^ using the gradients of the target to represent a localization map highlighting the main regions in the image for predicting the target is presented in session 5.

In each session, the experts sequentially analyzed the OCT images in a predetermined order. The reading environment of each reader remained constant throughout the sessions. An in-house tool specifically designed for this study facilitated a standardized evaluation process. In the session that included AI-assistance, the original OCT images were overlaid with a heatmap that could be easily switched on and off using a user-friendly on/off toggle. Experts considered both the AI-assistance results and original OCT findings, forming their individual judgments using a binary-point scale. The information provided in each reading session and the experimental screen are shown in Supplementary Fig. 1.

### Evaluation of the predicted model and statistical analysis

The performance of the experiment was measured using an AUROC score, which became the predictive accuracy of the algorithm. To compare AUROC score across reading sessions, we employed DeLong’s test^24^, a standard nonparametric method for assessing differences between correlated ROC curves and widely used in multi-reader designs^25^. In this study, DeLong’s test was applied to ROC curves generated by pooling the predictions of all readers within each session, rather than at the individual-reader level. Because this was an exploratory multi-session reader study, no formal multiple-comparison correction was applied. Session 5 served as the AI-assisted reference condition and, therefore, no pairwise comparison was performed for this session.

Statistical analysis was conducted using the difference in Fleiss’ kappa^26^. Fleiss’s kappa is an adaptation of Cohen’s kappa designed for scenarios involving three or more raters, making it particularly suitable for this reader study with multiple participants. Kruskal–Wallis and Chi-squared tests were used for comparisons between the distributions of datasets and agreement analyses. Statistical significance was set at *p* < 0.05, ensuring stringent criteria for determining the significance of the observed differences.

## Supplementary Information

Supplementary Information.

## Data Availability

All data generated or analyzed during this study are included in this published article.
