# Supplementary material for: Artificial intelligence based prediction of first recurrence in neovascular age related macular degeneration with validation by 19 experts
Source: Sci Rep. 2026 Jan 16;16:4440. doi: 10.1038/s41598-025-34480-8 (PMC12865193; doi:10.1038/s41598-025-34480-8)
Supplement: Supplementary file 1 — Supplementary Information. [file 41598_2025_34480_MOESM1_ESM.pdf]

Supplementary information files

AI-Driven First Recurrence Prediction in Neovascular Age-Related Macular Degeneration: A 19-Expert Validation Study

Boa Jang, BS; Chan Ho Lee, MD; Seung Jin Kim, BS; Chang Ki Yoon, MD, PhD; Un Chul Park, MD, PhD; Jinwook Choi, MD, PhD; Eun Kyoung Lee, MD, PhD; and Young-Gon Kim, PhD

Supplementary Figure 1. Details of each reading sessions, (**a**) Session 1 included optical coherence tomography (OCT) images at baseline; (**b**) Session 2 included OCT images at after the loading phase; (**c**) Session 3 included OCT images at both baseline and after the loading phase; (**d**) Session 4 included OCT images of both and additional clinical information, including age, gender, age-related macular degeneration (AMD) types, anti-vascular endothelial growth factor type, and visual acuity at baseline and after the loading phase; (**e**) Session 5 included all available information along with the results of AI-assistance.

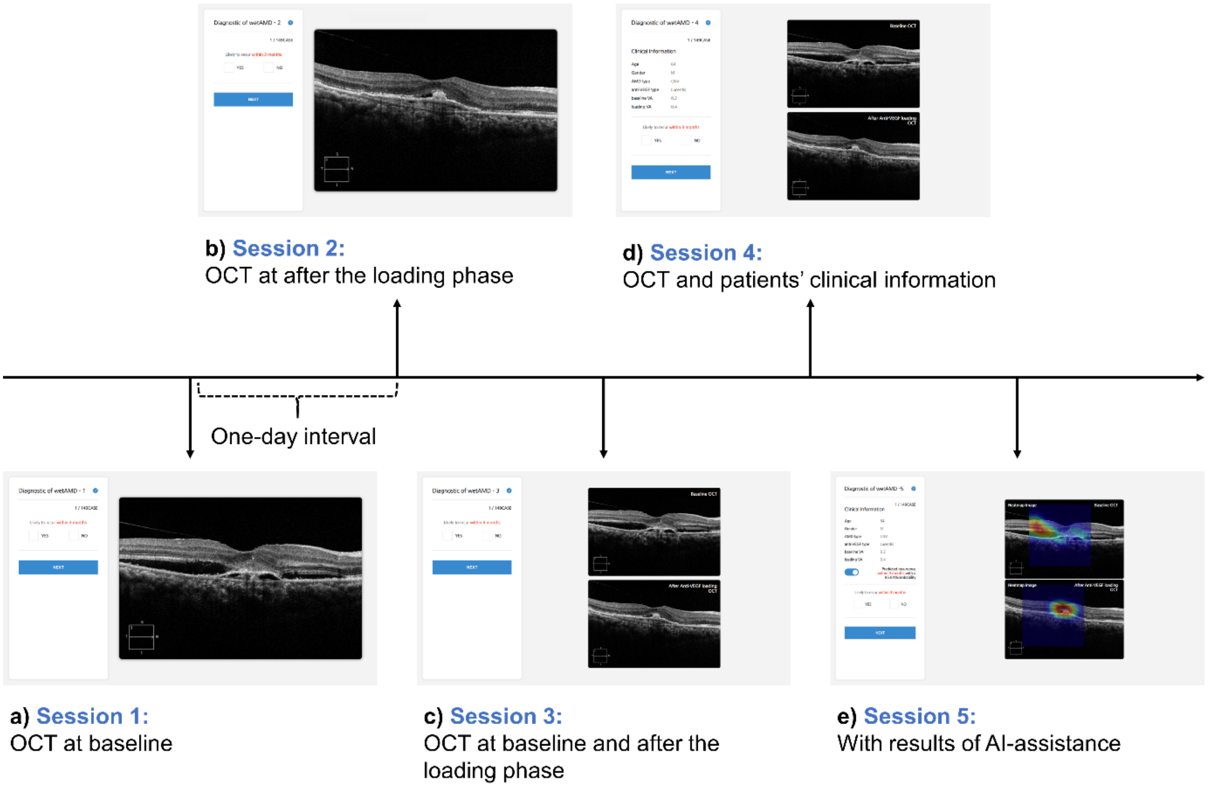

Supplementary Figure 2. Receiver operating characteristic curve plots of each session.

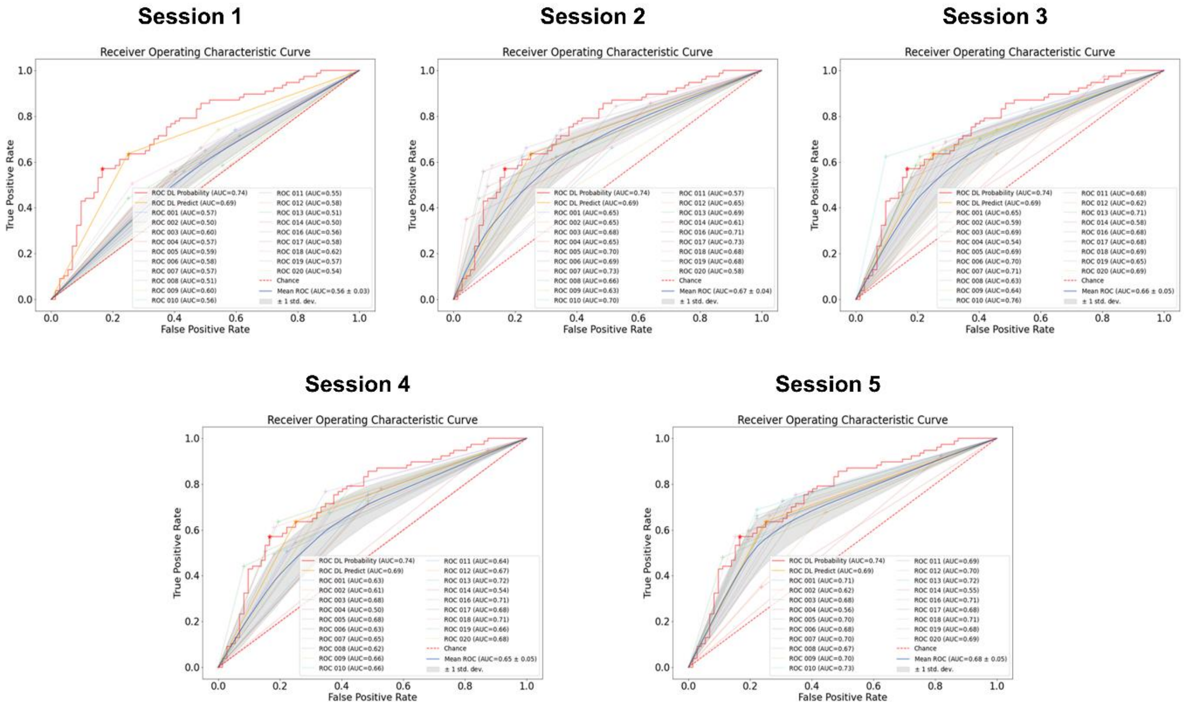

Supplementary Table 1. Number of ophthalmologists and grouping.

Variables	Retinal specialist ophthalmologists	Non-retinal specialist ophthalmologists	Total
**Number of readers**	9	10	19
**Age**	36.89 ± 5.49	33.30 ± 5.42	35.00 ± 5.61
**Gender**			
Male	9 (47.37)	6 (31.58)	15 (78.94)
Female	0	4 (21.05)	4 (21.05)
**Years of experience**			
Years	10.67 ± 6.80	6.50 ± 5.17	8.47 ± 6.20

Continuous variables are reported as mean value ± standard deviation. All other data are presented as numbers (percentages).

Supplementary Table 2. Detailed performance metrics of the AI-based CADx system.

	OCT at baseline	OCT after the loading phase	Ensemble (AI-based CADx)
**AUROC [95% CIs]**	0.600 [0.568-0.743]	0.725 [0.658-0.817]	0.744 [0.655-0.822]
**Accuracy**	0.572	0.679	0.698
**Sensitivity**	0.566	0.621	0.571
**Specificity**	0.578	0.742	0.833
**PPV**	0.599	0.725	0.786
**NPV**	0.557	0.649	0.645
**F1 Score**	0.573	0.665	0.622

OCT, optical coherence tomography; AUC, area under the curve; PPV, positive predictive value; NPV, negative predictive value.

Supplementary Table 3. Reliability of agreement among different readers in five reading sessions.

**Session**	**1**	**2**	**3**	**4**	**5**
Fleiss' kappa	0.224**	0.405	0.397*	0.369**	**0.480**
p-value	*0.001*	*0.161*	*0.017*	*0.006*	*Ref.*

Scoring Convention for Fleiss' Kappa: < 0 Poor agreement; 0.01 - 0.20 Slight agreement; 0.21 - 0.40 Fair agreement; 0.41 - 0.60 Moderate agreement; 0.61 - 0.80 Substantial agreement; 0.81 - 1.00 Almost perfect agreement.

p-values were calculated using a paired *t*-test. $^{*}p < .05$, $^{**}p < .01$, $^{***}p < .001$.

No *p*-value is provided for session 5 because it served as the AI-assisted reference session, and no direct comparison was performed.

Supplementary Table 4. Subgroup analysis with good agreement cases.

**Variables**	**Good agreement prediction with recurrence within 3 months**	**Good agreement prediction with recurrence after 3 months**	***p*-value**
**Number of samples**	46	51	
**Age**	70.9 ± 7.9	71.2 ± 8.1	*0.860**
**BCVA at baseline (logMAR)**	0.80 ± 0.55	0.67 ± 0.54	*0.261**
**nAMD subtype**			*0.618*†
Type 1 or 2 CNV	37 (80.4)	42 (82.4)	
PCV	4 (8.7)	6 (11.8)	
RAP (Type 3 CNV)	5 (10.9)	3 (5.9)	
**Anti-VEGF used for loading phase**			*0.122*†
Aflibercept	14 (30.4)	26 (51.0)	
Ranibizumab	27 (58.7)	21 (41.2)	
Bevacizumab	5 (10.9)	4 (7.8)	
**OCT parameters**			
**SRH at baseline**			*< 0.001*†
SRH (+)	37 (80.4)	6 (11.8)	
SRH (-)	9 (16.7)	45 (88.2)	
**SRF at baseline**			*0.470*†
SRF (+)	40 (87.0)	43 (84.3)	
SRF (-)	6 (13.0)	8 (15.7)	
**IRF at baseline**			*0.240*†
IRF (+)	20 (44.4)	18 (35.3)	
IRF (-)	25 (55.6)	33 (64.7)	
**HF at baseline**			*< 0.001*†
HF (+)	39 (84.8)	16 (31.4)	
HF (-)	7 (15.2)	35 (68.6)	
**HF after the loading phase**			*< 0.001*†
HF (+)	40 (87.0)	5 (9.8)	
HF (-)	6 (13.0)	46 (90.2))	

BCVA, best-corrected visual acuity; logMAR, logarithm of the minimum angle of resolution; nAMD, neovascular age-related macular degeneration; CNV, choroidal neovascularization; PCV, polypoidal choroidal vasculopathy; RAP, retinal angiomatous proliferation; anti-VEGF, anti-vascular endothelial growth factor; OCT, optical coherence tomography; SRH, subretinal hemorrhage; SRF, subretinal fluid; IRF, intraretinal fluid; HF, hyperreflective foci.

** Student independent t-test.*

† Chi-square test or Fisher exact test.

All data are reported as numbers (percentages) or mean value ± standard deviation.
